# Nonalcoholic steatohepatitis in hepatocarcinoma: new insights about its prognostic role in patients treated with lenvatinib

**DOI:** 10.1016/j.esmoop.2021.100330

**Published:** 2021-11-27

**Authors:** M. Rimini, M. Kudo, T. Tada, S. Shigeo, W. Kang, G. Suda, A. Jefremow, V. Burgio, M. Iavarone, R. Tortora, F. Marra, S. Lonardi, E. Tamburini, F. Piscaglia, G. Masi, G. Cabibbo, F.G. Foschi, M. Silletta, T. Kumada, H. Iwamoto, T. Aoki, M.J. Goh, N. Sakamoto, J. Siebler, A. Hiraoka, T. Niizeki, K. Ueshima, T. Sho, M. Atsukawa, M. Hirooka, K. Tsuji, T. Ishikawa, K. Takaguchi, K. Kariyama, E. Itobayashi, K. Tajiri, N. Shimada, H. Shibata, H. Ochi, S. Yasuda, H. Toyoda, S. Fukunishi, H. Ohama, K. Kawata, J. Tani, S. Nakamura, K. Nouso, A. Tsutsui, T. Nagano, T. Takaaki, N. Itokawa, T. Okubo, T. Arai, M. Imai, K. Joko, Y. Koizumi, Y. Hiasa, A. Cucchetti, F. Ratti, L. Aldrighetti, S. Cascinu, A. Casadei-Gardini

**Affiliations:** 1 Department of Oncology and Hematology, Division of Oncology, University of Modena and Reggio Emilia, Modena, Italy; 2 Department of Gastroenterology and Hepatology, Kindai University Faculty of Medicine, Higashi-osaka, Japan; 3 Department of Internal Medicine, Japanese Red Cross Himeji Hospital, Himeji, Japan; 4 Division of Gastroenterology, Department of Medicine, Kurume University School of Medicine, Kurume, Fukuoka, Japan; 5 Department of Medicine, Samsung Medical Center, Sungkyunkwan University School of Medicine, Seoul, Korea; 6 Department of Health Sciences and Technology, Samsung Advanced Institute for Health Sciences and Technology (SAIHST), Sungkyunkwan University, Seoul, Korea; 7 Department of Gastroenterology and Hepatology, Hokkaido University Graduate School of Medicine, Sapporo, Japan; 8 Department of Medicine 1, University Hospital Erlangen, Friedrich-Alexander-Universität Erlangen-Nuremberg, Erlangen, Germany; 9 Deutsches Zentrum Immuntherapie (DZI), Erlangen, Germany; 10 Department of Oncology, IRCCS San Raffaele Scientific Institute Hospital, Milan, Italy; 11 Fondazione IRCCS Ca' Granda Ospedale Maggiore Policlinico di Milano, Division of Gastroenterology and Hepatology, Milan, Italy; 12 Liver Unit, Department of Transplantation, Cardarelli Hospital, Naples, Italy; 13 Department of Experimental and Clinical Medicine, University of Florence, Florence, Italy; 14 Medical Oncology Unit 3, Department of Oncology, Veneto Institute of Oncology IOV-IRCCS, Padua, Italy; 15 Department of Medical Oncology, Card. G. Panico Hospital of Tricase, Tricase, Italy; 16 Division of Internal Medicine, IRCCS Azienda Ospedaliero-Universitaria di Bologna, Bologna, Italy; 17 Unit of Medical Oncology, Pisa University Hospital, Pisa, Italy; 18 Section of Gastroenterology & Hepatology, Department of Health Promotion, Mother and Child Care, Internal Medicine and Medical Specialties, PROMISE, University of Palermo, Palermo, Italy; 19 Azienda Unità Sanitaria della Romagna, Ospedale degli Infermi, Faenza, Italy; 20 Medical Oncology Unit, University Campus Bio-Medico, Rome, Italy; 21 Faculty of Nursing, Gifu Kyoritsu University, Ogaki, Japan; 22 Gastroenterology Center, Ehime Prefectural Central Hospital, Matsuyama, Japan; 23 Division of Gastroenterology and Hepatology, Department of Internal Medicine, Nippon Medical School, Tokyo, Japan; 24 Department of Gastroenterology and Metabology, Ehime University Graduate School of Medicine, Matsuyama, Japan; 25 Center of Gastroenterology, Teine Keijinkai Hospital, Sapporo, Japan; 26 Department of Gastroenterology, Saiseikai Niigata Hospital, Niigata, Japan; 27 Department of Hepatology, Kagawa Prefectural Central Hospital, Takamatsu, Japan; 28 Department of Gastroenterology, Okayama City Hospital, Okayama, Japan; 29 Department of Gastroenterology, Asahi General Hospital, Asahi, Japan; 30 Department of Gastroenterology, Toyama University Hospital, Toyama, Japan; 31 Division of Gastroenterology and Hepatology, Otakanomori Hospital, Kashiwa, Japan; 32 Department of Gastroenterology, Tokushima Prefectural Central Hospital, Tokushima, Japan; 33 Hepato-biliary Center, Matsuyama Red Cross Hospital, Matsuyama, Japan; 34 Department of Gastroenterology and Hepatology, Ogaki Municipal Hospital, Ogaki, Japan; 35 Second Department of Internal Medicine, Osaka Medical College, Takatsuki, Japan; 36 Hepatology Division, Department of Internal Medicine, Hamamatsu University School of Medicine, Hamamatsu, Japan; 37 Department of Gastroenterology and Neurology, Kagawa University School of Medicine, Kagawa, Japan; 38 Department of Medical and Surgical Sciences-DIMEC, Alma Mater Studiorum – University of Bologna, Bologna, Italy; 39 Department of Surgery, Morgagni – Pierantoni Hospital, Forlì, Italy; 40 Hepatobiliary Surgery Division, IRCCS San Raffaele Scientific Institute, Milan, Italy; 41 Vita-Salute San Raffaele University, Milan, Italy

**Keywords:** advanced hepatocarcinoma, nonalcoholic steatohepatitis, lenvatinib, sorafenib, immunotherapy, atezolizumab, bevacizumab, hepatitis C, hepatocellular carcinoma

## Abstract

**Background:**

Hepatocellular carcinoma (HCC) treatment remains a big challenge in the field of oncology. The liver disease (viral or not viral) underlying HCC turned out to be crucial in determining the biologic behavior of the tumor, including its response to treatment. The aim of this analysis was to investigate the role of the etiology of the underlying liver disease in survival outcomes.

**Patients and methods:**

We conducted a multicenter retrospective study on a large cohort of patients treated with lenvatinib as first-line therapy for advanced HCC from both Eastern and Western institutions. Univariate and multivariate analyses were performed.

**Results:**

Among the 1232 lenvatinib-treated HCC patients, 453 (36.8%) were hepatitis C virus positive, 268 hepatitis B virus positive (21.8%), 236 nonalcoholic steatohepatitis (NASH) correlate (19.2%) and 275 had other etiologies (22.3%). The median progression-free survival (mPFS) was 6.2 months [95% confidence interval (CI) 5.9-6.7 months] and the median overall survival (mOS) was 15.8 months (95% CI 14.9-17.2 months). In the univariate analysis for OS NASH-HCC was associated with longer mOS [22.2 versus 15.1 months; hazard ratio (HR) 0.69; 95% CI 0.56-0.85; *P* = 0.0006]. In the univariate analysis for PFS NASH-HCC was associated with longer mPFS (7.5 versus 6.5 months; HR 0.84; 95% CI 0.71-0.99; *P* = 0.0436). The multivariate analysis confirmed NASH-HCC (HR 0.64; 95% CI 0.48-0.86; *P* = 0.0028) as an independent prognostic factor for OS, along with albumin–bilirubin (ALBI) grade, extrahepatic spread, neutrophil-to-lymphocyte ratio, portal vein thrombosis, Eastern Cooperative Oncology Group (ECOG) performance status and alpha-fetoprotein. An interaction test was performed between sorafenib and lenvatinib cohorts and the results highlighted the positive predictive role of NASH in favor of the lenvatinib arm (*P* = 0.0047).

**Conclusion:**

NASH has been identified as an independent prognostic factor in a large cohort of patients with advanced HCC treated with lenvatinib, thereby suggesting the role of the etiology in the selection of patients for tyrosine kinase treatment. If validated, this result could provide new insights useful to improve the management of these patients.

## Highlights

• Evidence supported the idea that etiology could sustain a crucial role in biological behavior.
• NASH constitutes one of the more important risk factors for hepatocarcinoma, and its incidence is increasing very fast.
• We performed an analysis in patients treated with lenvatinib as the first-line therapy.
• NASH was found to be an independent prognostic factor.

## Introduction

Hepatocellular carcinoma (HCC) treatment constitutes a big challenge in the field of oncology, due to the complexity of its pathogenesis and the heterogeneity of etiology. Potentially curative treatments, including liver transplantation and tumor resection or ablation, are limited to early-stage tumor,^1^^,^^2^ whereas palliative treatments, including embolization and medical drugs, are prerogative of the intermediate and advanced stages. In the recent years the panorama of therapeutic options for patients with advanced disease not suitable for locoregional treatment has dramatically increased, with the approval of new drugs in this setting. First, the multikinase inhibitor sorafenib was approved for the first-line treatment of patients with advanced HCC^3^^,^^4^; consequently, regorafenib,^5^ cabozantinib^6^ and ramucirumab^7^^,^^8^ were inserted as further treatments after sorafenib failure in the armamentarium against advanced HCC. Recently, the results of the REFLECT trial confirmed the noninferiority in terms of overall survival (OS) of another multikinase inhibitor lenvatinib compared with sorafenib^9^; but demonstrated a superiority in terms of progression-free survival (PFS).^9^ Moreover, the REFLECT trial highlighted similar baseline scores on the European Organisation for Research and Treatment of Cancer Core Quality of Life questionnaire (EORTC QLQ)-C30 and EORTC QLQ-HCC18 health questionnaires in the two arms, but analysis of time to clinically meaningful deterioration showed that role functioning, pain and diarrhea were experienced earlier in the group of patients treated with sorafenib compared with those receiving lenvatinib.^9^ Besides multikinase inhibitors, in the past years another class of drug has been introduced in the advanced HCC setting armamentarium: immunotherapy. The two programmed cell death protein 1 (PD-1)-directed antibodies nivolumab and pembrolizumab failed to reach the primary endpoint in the single-agent phase III trials.^1^^,^^10^^,^^11^, ^12^, ^13^ Subsequently, the phase III IMBRAVE-150 trial investigating the combination of the anti-programmed death-ligand 1 (PD-L1) atezolizumab with the anti-VEGF bevacizumab demonstrated an advantage over sorafenib in terms of both OS and PFS, thus leading to the approval of the combination as first-line new standard of care for advanced HCC.^14^ A recent press release reported the results from the phase III randomized trial KEYNOTE-394, which investigated pembrolizumab versus placebo in advanced HCC patients pretreated with sorafenib or oxaliplatin-based chemotherapy. The preliminary results found that pembrolizumab statistically improved OS compared with placebo, thus meeting the primary endpoint of the study. In addition, the study met its secondary endpoints of PFS and objective response rate, and no new adverse events (AEs) were reported.^15^ Because of the extreme complexity of the HCC setting, which includes different situations depending on the underlying liver disease, the researchers have made their efforts toward revealing the possible role of the HCC etiology in affecting response rate and survival outcomes. More specifically, it has been recently hypothesized that the viral and/or not viral etiology of the underlying liver disease could determine different hepatic microenvironments with a consequent different responsiveness to determinate treatments.^16^ New interesting insights have been recently provided about the mechanisms of nonalcoholic steatohepatitis (NASH), mainly because of a significant increase in its incidence in the Western countries.^1^^,^^17^ NASH is defined as ≥5% liver steatosis, including inflammation and injury to hepatocytes frequently associated with fibrosis, and is considered an evolution of nonalcoholic fatty liver disease (NAFLD).^18^ NAFLD is a chronic liver disorder that encompasses a heterogeneous spectrum of disease. Accumulating evidence supports an association between NAFLD and components of metabolic syndrome, because the main causes of these two conditions, including obesity, excessive intake of simple sugars and physical inactivity, are the same. Because metabolic syndrome can be defined in different ways, NAFLD might be considered a direct predictor of this disease, thus leading to a difficulty in separating the concepts, which are frequently used as a part of the same condition.

For instance, sorafenib was demonstrated in several reports to provide the best outcomes in HCC on a background of hepatitis C virus-related cirrhosis. Innate and adaptative immune-cell activation in combination with the endoplasmic reticulum stress and increased release of metabolites in patients with NASH are hypothesized to trigger the necro-inflammation of hepatocytes with a consequent fibrotic regeneration, and increased risk of HCC.^19^, ^20^, ^21^, ^22^, ^23^, ^24^, ^25^ Starting from these premises, we performed a subgroup analysis on a large population of advanced HCC patients treated with lenvatinib as first-line therapy, to investigate the role of the etiology of the underlying liver disease on survival outcomes.

## Methods

This is a multicenter retrospective study of prospectively enrolled patients treated with lenvatinib as first line for Barcelona Clinic Liver Centre (BCLC) stage B or C HCC, who were deemed not eligible for first or retreatment with surgical or locoregional therapies. The population included Eastern and Western populations from Japan, Korea, Germany and Italy between July 2010 and May 2021.

Eligible patients had HCC diagnosis histologically and/or clinically confirmed in accordance with international guidelines and none of them received previous systemic therapy.

Eligibility criteria were Eastern Cooperative Oncology Group (ECOG) performance status score ≤2; Child–Pugh liver function class ≤B7; adequate hematologic function (platelet count ≥60 × 10^9^/l; hemoglobin ≥8.5 g/dl and prothrombin time international normalized ratio ≤2.3 or prothrombin time ≤6 s above control); adequate hepatic function [albumin ≥2.8 g/dl; total bilirubin ≤3 mg/dl (51.3 μmol/l); alanine aminotransferase and aspartate aminotransferase ≤5 times the upper limit of the normal range] and adequate renal function (serum creatinine ≤1.5 times the upper limit of the normal range).

We used European Association for the Study of the Liver–European Association for the Study of Diabetes–European Association for the Study of Obesity (EASL–EASD–EASO) Clinical Practice Guidelines for defining cases as ‘NASH-related HCC’.^24^ NASH-related HCC' was defined based on the presence of these parameters:

• Presence of steatosis in >5% of hepatocytes according to histological analysis.
• Patients without a history of alcohol abuse (30 g for men and 20 g for women).
• Hepatitis B and C negativity.

Lenvatinib was administered as described in the REFLECT trial,^9^ thus patients received 12 mg if baseline bodyweight was ≥60 kg or 8 mg if baseline bodyweight was <60 kg; lenvatinib was given orally once a day.

Follow-up consisted of a computed tomography/magnetic resonance imaging scan every 8 weeks or as clinically indicated. Tumor response was evaluated in accordance with modified RECIST.

Patients continued lenvatinib if they had clinical benefit as judged by the physician in charge, or until unacceptable toxicity.

Treatment interruptions and dose reductions were allowed to manage AEs.

All patients provided written informed consent before their enrolment in the study. This study was approved by ethics committee at each center, complied with the provisions of the Good Clinical Practice guidelines and the Declaration of Helsinki and local laws and fulfilled the Regulation (EU) 2016/679 of the European Parliament and of the Council of 27 April 2016 on the protection of natural persons with regard to the processing of personal data.

## Results

### Lenvatinib cohort

Among the 1232 lenvatinib-treated HCC patients of the study cohort, 453 (36.8%) were hepatitis C virus positive, 268 hepatitis B virus positive (21.8%), 236 NASH related (19.2%) and 275 with other etiologies (22.3%). Baseline clinicopathologic and laboratory characteristics are summarized in Table 1.

**Table 1:** Patient's characteristics at baseline

Parameters	All Population	NASH correlate	No NASH correlate	*P* value
Age (years), median (range)	75 (25-91)	76 (45-91)	73 (25-89)	0.41
Gender				0.13
Female	21.7	25.4	20.8	
Male	78.3	74.6	79.2	
ECOG PS				0.92
0	82.0	81.8	82.0	
>0	18.0	18.2	18.0	
Child–Turcotte–Pugh score				
A	88.2	88.1	88.2	1.00
B	11.8	11.9	11.8	1.00
BCLC stage				0.66
B	44.2	42.8	44.6	
C	55.8	57.2	55.4	
Portal vein thrombosis				0.91
Yes	21.4	22.2	21.2	
No	78.6	77.8	78.8	
ALBI grade				0.04
1	89.3	91.4	88.8	
2	10.7	8.6	11.2	
Neutrophil-to-lymphocyte ratio				0.13
<3	63.4	68.2	62.3	
>3	36.6	31.8	37.7	
AFP				0.0002
<400	68.8	78.8	66.6	
>400	31.2	21.2	33.4	
Subsequent therapy				0.13
TACE		22.6	18.1	
Immunotherapy		3.1	6.8	
Sorafenib		23.3	18.1	
Other treatments		3.8	4.8	
No treatment		47.2	52.2	

AFP, alpha-fetoprotein; ALBI, albumin–bilirubin; BCLC, Barcelona Clinic Liver Centre (staging); ECOG PS, Eastern Cooperative Oncology Group performance status; NASH, nonalcoholic steatohepatitis; TACE, transarterial chemoembolization.

Patients with or without NASH differed for albumin–bilirubin (ALBI) grade and alpha-fetoprotein.

The median PFS (mPFS) was 6.2 months [95% confidence interval (CI) 5.9-6.7 months] and the median OS (mOS) was 15.8 months (95% CI 14.9-17.2 months).

**Figure 1:**
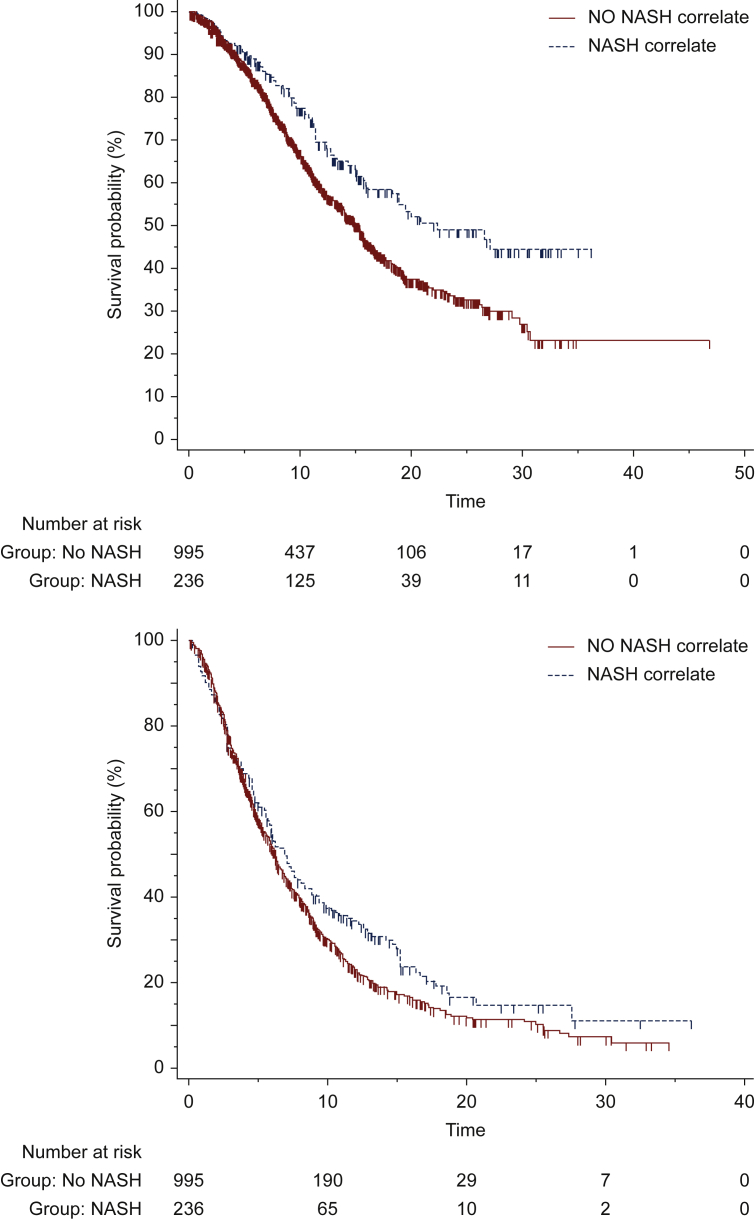
Kaplan–Meier curves for (A) overall survival to lenvatinib and (B) progression-free survival to lenvatinib in patients with nonalcoholic steatohepatitis (NASH)-related hepatocellular carcinoma (HCC) and no NASH-related HCC.

In the univariate analysis for OS NASH-HCC was associated with longer mOS [22.2 versus 15.1 months; hazard ratio (HR) 0.69; 95% CI 0.56-0.85; *P* = 0.0006; Figure 1A]. In the univariate analysis for PFS NASH-HCC was associated with longer mPFS (7.5 versus 6.5 months; HR 0.84; 95% CI 0.71-0.99; *P* = 0.0436; Figure 1B).

In addition, Child–Pugh B versus A (HR 2.22; 95% CI 1.63-3.02; *P* < 0.0001), ALBI grade 2 versus 1 (HR 3.33; 95% CI 2.37-4.69; *P* < 0.0001), BCLC C versus B (HR 1.65; 95% CI 1.39-1.97; *P* < 0.0001), extra hepatic spread yes versus no (HR 1.68; 95% CI, 1.38-2.03; *P* < 0.0001), neutrophil-to-lymphocyte ratio ≥3 versus <3 (HR 1.78; 95% CI 1.45-2.20; *P* < 0.0001), portal vein thrombosis yes versus no (HR 1.63; 95% CI 1.19-2.22; *P* = 0.0019), ECOG >0 versus 0 (HR 1.55; 95% CI 1.16-2.07; *P* = 0.0025), alpha-fetoprotein ≥400 versus <400 ng/ml (HR 1.47; 95% CI 1.14-1.90; *P* = 0.0024) and hepatitis B virus-positive status (HR 1.28; 95% CI 1.03-1.58; *P* = 0.0253) were correlated with poor prognosis. Other etiologies were not associated with positive clinical outcome (Table 2).

**Table 2:** Univariate and multivariate analyses

Variable	Univariate analysis		Multivariate analysis	
Gender				
Male	1			
Female	1.06 (0.85-1.32)	0.5776		
Child–Turcotte–Pugh score				
A	1		1	
B	2.22 (1.63-3.02)	**<0.0001**	1.07 (0.74-1.55)	0.6893
ALBI				
1	1		1	
2	3.33 (2.37-4.69)	**<0.0001**	1.80 (1.25-2.58)	**0.0013**
BCLC				
B	1		1	
C	1.65 (1.39-1.97)	**<0.0001**	0.99 (0.72-1.35)	0.9373
Extra hepatic spread				
No	1		1	
Yes	1.68 (1.38-2.03)	**<0.0001**	1.65 (1.25-2.18)	**0.0003**
Neutrophil-to-lymphocyte ratio				
<3	1		1	
>3	1.78 (1.45-2.20)	**<0.0001**	1.59 (1.30-1.94)	**<0.0001**
Portal vein thrombosis				
No	1		1	
Yes	1.63 (1.19-2.22)	**0.0019**	1.32 (1.02-1.71)	**0.0342**
ECOG				
0	1		1	
>0	1.47 (1.14-1.90)	**0.0024**	1.33 (1.00-1.75)	**0.0458**
AFP				
<400	1		1	
>400	1.64 (1.34-2.01)	**<0.0001**	1.27 (1.02-1.58)	**0.0306**
NASH				
Not correlated	1		1	
Correlated	0.69 (0.56-0.85)	**0.0006**	0.64 (0.48-0.86)	**0.0028**
HCV				
Positive	1			
Negative	0.99 (0.83-1.19)	0.9983		
HBV				
Negative	1		1	
Positive	1.28 (1.03-1.58)	**0.0253**	1.22 (0.96-1.55)	0.0963
Other etiology				
Yes	1			
No	0.86 (0.69-1.07)	0.1919		

AFP, alpha-fetoprotein; ALBI, albumin–bilirubin; BCLC, Barcelona Clinic Liver Centre (staging); ECOG PS, Eastern Cooperative Oncology Group performance status; HBV, hepatitis B virus; HCV, hepatitis C virus; NASH, nonalcoholic steatohepatitis; TACE, transarterial chemoembolization.

Bold indicates positive results.

Following adjustment for clinical covariates positive in univariate analysis, multivariate analysis confirmed NASH-HCC (HR 0.64; 95% CI 0.48-0.86; *P* = 0.0028) as independent favorable prognostic factors for OS, with ALBI grade 1, absence of extrahepatic spread, neutrophil-to-lymphocyte ratio <3, absence of portal vein thrombosis, ECOG PS 0 and alpha-fetoprotein <400 (Table 2).

Different from mOS and mPFS, NASH etiology was not associated with a different rate of tumor response or occurrence of AEs. In fact, no differences were found in terms of percentage of response rate (partial response plus complete response) at the first computed tomography re-evaluation (35.1% in patients with NASH and 34.3% in patients without NASH, *P* = 0.81). By considering patients who experienced a response rate, different outcomes in terms of mPFS and mOS were observed between patients with NASH and patients without NASH (mPFS 13.6 versus 10.8 months, *P* = 0.02; mOS 30.6 versus 21.6 months, *P* = 0.013, respectively).

AEs of any grade were reported in 1181 patients (95.9%); 15.4%, 12.6% and 9.1% of patients reported hand–foot skin toxicity, hypertension and diarrhea of grade 3/4, respectively. Grade 3/4 AEs were similar between patients with or without NASH, with the only difference found in terms of hypertension (18.9% versus 12.3%; *P* = 0.001).

### Sorafenib cohort and predictive role of NASH

To test the predictive role of NASH we evaluated the prognostic impact in terms of OS in a cohort treated with sorafenib. A total of 483 sorafenib-treated HCC patients were included, 116 (24.0%) were NASH related and 367 (76.0%) were not NASH related. mOS was 12.0 months (95% CI 10.8-36.3 months).

In the univariate analysis for OS NASH-HCC was not associated with longer mOS (10.2 versus 12.3 months; HR 1.06; 95% CI 0.83-1.34; *P* = 0.6305).

Interaction test highlighted the positive predictive role of NASH in favor of the lenvatinib arm (*P* = 0.0047).

## Discussion

To the best of our knowledge, this is the first analysis defining the prognostic role of NASH-HCC in advanced HCC patients treated with lenvatinib. Both OS and PFS were statistically improved in the group of patients with NASH compared with those without NASH in our analysis. In particular, an mOS of 22.2 months was achieved for lenvatinib in patients with NASH, which is an impressive result considering the advanced HCC setting. Even if the population we considered included a significant proportion of HCC patients with BCLC B (BCLB 44.2% versus BCLC C 55.8%), which could have influenced our survival results, no statistical differences in terms of BCLC stage have been revealed between the group of patients with NASH-related HCC and those with no NASH-related HCC (*P* = 0.66). Moreover, even if considering a population with good prognosis a clinical factor, the survival revealed in NASH patients remains impressive. Despite the well-known limits of a transversal comparison between trials with different designs and samples, it is notable to focus on the mOS of 13.6 months reported in the lenvatinib noninferiority registration study.^9^ In our analysis, although no differences were reported between the two groups of patients in terms of response rate, the PFS analysis confirmed a statistically significant advantage in patients with NASH compared with those without NASH. This result is noteworthy, mainly if compared with the data derived from the *post hoc* analysis of the REFLECT trial.^26^ Results highlighted no difference in subsequent therapies and response rate between patients with and without NASH in our study; however, the respondents had a different outcome in mPFS and mOS, and this aspect could explain the discrepancy in the lack of correlation between NASH response and improved mOS.

Interestingly, clinicopathologic and laboratory characteristics at the baseline were similar in patients with NASH-driven HCC and patients with no NASH-driven HCC, with the exception of the ALBI grade and AFP. In the univariate analysis, both ALBI grade and AFP had a prognostic impact on our cohort of patients, as also reported in several other papers.^26^, ^27^, ^28^ Differences in terms of AFP and ALBI scores could not be ignored, because the prognostic value of both these parameters could potentially constitute a kind of selection bias when comparing the survival outcomes of the two groups of patients we considered (NASH-related HCC versus no NASH-related HCC). For this reason, and because both ALBI score and AFP were prognostic predictors in univariate analysis, we included both the parameters in the multivariate analysis. Interestingly, even this way, multivariate analysis also confirmed NASH-HCC to be a positive prognostic factor for OS in HCC patients treated with lenvatinib.

Our special interest in NASH-driven HCC derived from three starting concepts: first, the lack of biomarkers to stratify patients likely to respond to treatment in an advanced HCC setting; second, the emerging role of NASH in the HCC epidemiology and third the recent evidence of a lack of efficacy of immunotherapy in patients with NASH.

NASH is characterized by severe hepatocellular injury and constitutes the inflammatory consequence of NAFLD. This condition is strongly associated with metabolic syndrome, which includes obesity, type II diabetes mellitus, dyslipidemia and hypertension.^29^^,^^30^ The progressive rise of obesity prevalence worldwide in recent years has therefore lead to a dramatic increase in NAFLD incidence, with a consequent growth of NASH-driven HCC and cancer-related death.^29^ Moreover, modelling studies report that the prevalence of NASH and NASH-related HCC will continue to rise internationally in the immediate future.^31^^,^^32^ The underlying pathways leading to the transformation of NASH in HCC are not completely understood, but inflammatory status of microenvironment, oxidative stress and aberrant liver regeneration process are surely involved.^33^ Because of its peculiar interplay with the immunological system, NASH-related HCC has become a focus of interest in the oncologic field in the immunotherapy era.^34^, ^35^, ^36^ Pfister and collaborators reported evidence from both preclinical and clinical models, with all pointing toward the hypothesis that NASH-HCC might be less responsive to immunotherapy, probably due to a peculiar phenotype of T cells.^16^ By contrast, administration of anti-PD-1 drugs increased the number and size of HCC nodules in preclinical models, thereby suggesting a detrimental effect of CD8+ T cells rather than an invigorating effect on immune surveillance in this pathological setting.^16^ Furthermore, they performed a meta-analysis of three large and heterogeneous (first and second line, single-agent and combination immunotherapy) randomized controlled phase III clinical trials with immunotherapy. Their analysis highlighted the positive impact in terms of OS in patients with hepatis B or C; by contrast, no impact was noted in patients with HCC of nonviral etiology.^16^ Finally, the authors investigated a cohort of 130 patients with HCC, including 13 patients with NAFLD and 117 patients with HCC related to other etiologies. Patients with NAFLD experienced a shorter mOS with immunotherapy. These results were validated on a further cohort of 118 HCC patients treated with anti-PD-1/PD-L1 therapy.

These results constitute a rationale for stratifying patients with advanced HCC according to the underlying liver disease. Our results parallel those of the aforementioned study: NASH-related HCC patients, who seem to poorly respond to immunotherapy, might benefit from treatment with lenvatinib, thus suggesting a prognostic role of etiology in advanced HCC patients treated with lenvatinib.

Another important aspect revealed by our analysis concern the predictive role of NASH in patients treated with lenvatinib compared with patients treated with sorafenib. To the best of our knowledge, for the first time NASH-related etiology of HCC predicted response to lenvatinib treatment. This result is of special interest because it adds an important piece to the puzzle by delineating a fundamental role of the etiology not only in the carcinogenesis process and in cancer prognosis, but also in the response to specific therapeutic strategies. In the future, once validated with prospective cohorts of HCC patients, our results could lead to the definition of etiology as a stratification factor. In the evolving scenario of advanced HCC treatments, the definition of solid stratification factors may constitute a crucial tool to identify patients likely to benefit from one treatment rather than another (for example, TKI or immunotherapy), thus improving the management of these patients in clinical practice. Another insight concerns the safety profile: the present analysis showed that patients with and without NASH experienced almost the same incidence of AEs, apart from hypertension, which occurred more frequently in NASH-related HCC patients. In a previous work, patients who developed hypertension were shown to be associated with a significantly longer survival compared with those who did not experience it.^37^ Starting from this finding, we could be hypothesized as association between the better survival outcome revealed and the occurrence of hypertension in NASH patients.

Nevertheless, hypertension is included in the spectrum of symptoms of metabolic syndrome, thus making it difficult to elucidate the origin of hypertension in our patients, whether iatrogenic or not.

Our work presents some limitations. First, we performed a retrospective observational study on a large population, and we could not exclude a selection bias. Second, the multinational nature of the analysis did not allow for centralization of imaging, and the criteria for treatment and re-evaluation were consistent with the clinical practice of each center, and in accordance with national guidelines.

Nevertheless, this analysis was conducted on a sample of patients from different countries, including patients from both Asian and Western countries, thus constituting one of the largest real-life collection of data from patients with advanced HCC treated with lenvatinib.

In conclusion, our results provide new insights into the advanced HCC setting, suggesting a crucial role of the etiology of liver disease in identifying patients more likely to respond to a first-line treatment with lenvatinib. Collection of new evidence to validate our findings remains mandatory to confirm the role of etiology in stratifying patients to optimize treatment strategies in such a difficult setting.
